# Nutritional outcomes between different techniques of intradialytic amino acid replacement: a randomized controlled trial

**DOI:** 10.1093/ckj/sfae361

**Published:** 2024-11-18

**Authors:** Sophon Dumrongsukit, Khajohn Tiranathanagul, Pagaporn Asavapujanamanee, Kamonchanok Metta, Somchai Eiam-Ong, Piyawan Kittiskulnam

**Affiliations:** Division of Nephrology, Department of Medicine, Faculty of Medicine, Chulalongkorn University, Bangkok, Thailand; Division of Nephrology, Department of Medicine, Faculty of Medicine, Chulalongkorn University, Bangkok, Thailand; Benjakitti-MDCU Hemodialysis Center, Benjakitti Park Hospital, Bangkok, Thailand; Division of Nephrology, Department of Medicine, Faculty of Medicine, Chulalongkorn University, Bangkok, Thailand; Division of Nephrology, Department of Medicine, Faculty of Medicine, Chulalongkorn University, Bangkok, Thailand; Division of Nephrology, Department of Medicine, Faculty of Medicine, Chulalongkorn University, Bangkok, Thailand; Division of Internal Medicine-Nephrology, Department of Medicine, Faculty of Medicine, Chulalongkorn University and King Chulalongkorn Memorial Hospital, Thai Red Cross Society, Bangkok, Thailand

**Keywords:** albumin, amino acid, haemodialysis, malnutrition, parenteral nutrition

## Abstract

**Background:**

Amino acid (AA) depletion during dialysis deteriorates the protein-energy status of haemodialysis (HD) patients. This study aimed to determine whether intradialytic amino acid (IDAA) replacement by continuous infusion versus acute load could provide better nutritional outcomes.

**Methods:**

HD patients with mild protein-energy wasting, defined as a serum albumin level of 3.5–3.9 mg/dl despite 7-point subjective global assessment in category A or a malnutrition inflammation score ≤5, were randomly assigned to receive IDAA by continuous infusion or acute load for 3 months. In continuous infusion (*n* = 24), 50% glucose followed by 7.2% branched-chain enriched AA solution were instilled in the first 15 minutes after HD initiation with high-flux dialyser through the end of the session. Similar parenteral nutrition compositions containing the same total amount of glucose and AA were rapidly added into the venous drip chamber within the last hour of HD in the acute load group (*n* = 24). The primary outcome was the change in serum albumin level. Secondary outcomes were changes in muscle parameters and plasma as well as dialysate AA concentrations.

**Results:**

The mean age of patients was 68.9 ± 12.7 years and the average body mass index was 22.8 ± 4.4 kg/m^2^ with 45.8% being men. After 3 months, serum albumin levels were significantly elevated in continuous infusion (*P* = .001) whereas it was unchanged in the acute load (*P* = .13). Despite comparable energy and protein intake, total body muscle mass was also increased in the continuous infusion group at 3 months (*P* = .03) compared with no significant change in the acute load group (*P* = .45). The amount of AA loss into the dialysate was similar between the two groups (*P* = .17). At post-dialysis, most plasma essential and non-essential AA levels were significantly lower in patients receiving continuous infusion than acute load, while branched-chain AA concentrations including leucine (*P* = .61) and valine (*P* = .09) were comparable between the two groups. Despite enhancing muscle mass in continuous infusion, handgrip strength and gait speed were unaltered in both techniques of IDAA replacement.

**Conclusions:**

IDAA using continuous infusion appears to be superior to acute load in terms of serum albumin and muscle mass improvement. The impact of IDAA on hard clinical outcomes may require larger scale with a longer period of study (TCTR20230401003).

KEY LEARNING POINTS **What was known:**

- Amino acid (AA) depletion during dialysis deteriorates protein-energy status of haemodialysis patients.
- Currently there is no consensus on how best to deliver parenteral nutrition–containing AAs during dialysis.

**This study adds:**

- The continuous infusion technique of intradialytic AA replacement appears to be superior to acute load in terms of improvement in serum albumin level and muscle quantity.

**Potential impact:**

- Continuous infusion may be a preferred method for improving nutritional outcomes among dialysis patients requiring intradialytic AA replacement.

## INTRODUCTION

Protein-energy wasting (PEW), defined as a loss of body protein mass and fuel reserves, is common among end-stage kidney disease (ESKD) patients undergoing haemodialysis (HD) and has negative impact on patients’ prognosis and survival [1]. Although removal of uraemic toxins by dialysis attenuates anorexic symptoms, it promotes the occurrence of PEW through a significant loss of amino acids (AAs) into the dialysate, metabolic acidosis, inflammation and insulin resistance [2]. Data analysis from the Hemodialysis (HEMO) Study indicated that overall dietary protein and energy intakes are lower than the values recommended for patients undergoing HD [3]. Reduced nutrient intake combined with a loss of AAs into the dialysate deteriorates the nutritional status of HD patients over time. Former experimental studies revealed that intradialytic protein catabolism occurred more often than that of concurrent synthesis, resulting in a profound catabolic stress on whole-body and muscle protein homeostasis [4, 5]. Unlike a maintenance of equilibrium between protein catabolism and anabolism in well-nourished HD patients, these adaptive responses tend toward inequivalent balance even among HD patients with a pre-existing mild degree of PEW [6]. Due to the ineffective attempts to increase protein intake by either the oral or enteral route, exogenous intradialytic amino acid (IDAA) administered parenterally during the dialysis session has been widely employed as an important strategy to mitigate worsening of PEW in the HD population [7, 8].

There are certain variations in practice patterns of IDAA replacement among dialysis facilities for improving nutritional outcomes. In order to maximize the retention of infused AAs, a parenteral solution that was rapidly instilled using an infusion pump into the drip chamber of the venous site of dialyser near the end or immediately after the dialysis session has been employed [9, 10]. Nevertheless, such a procedure may increase the risk for fluid overload, require additional time to operate and cause adverse events from the rapid transfusion rate. Given that the dialysis process generally yields a net protein breakdown, a continuous infusion of IDAA replacement throughout the entire session has also been proposed to reduce procedure-related catabolic events at the initial onset. An earlier study demonstrated that plasma concentrations of most AAs were significantly increased by a continuous infusion of IDAA at a constant rate starting from the beginning of dialysis without differences in hourly losses of AAs [11]. On the other hand, a few observational studies showed that post-dialysis plasma AA levels were markedly reduced when using a continuous infusion of IDAA at the start of the dialysis session and the total amount of AAs lost into spent dialysate was higher than without AA replacement [12, 13]. Owing to the various techniques used for replacement, the effectiveness of IDAA for treatment of PEW reported by the updated meta-analysis remains inconclusive [14].

Apart from AAs as nutrient loss during each dialysis session, an accompanying energy deficit of 200–480 kcal should be taken into account [15]. Furthermore, the additional caloric supply could enhance the nitrogen balance and delivery of AAs to the muscle compartment [16]. A recent well-controlled study also indicated that intradialytic parenteral energy administration helped improve protein-energy status as well as the plasma AA profile among HD patients with PEW [17].

Prior studies regarding IDAA infusion techniques on beneficial nutritional outcomes are scarce. Most of them were lacking concurrent analysis of visceral and structural proteins along with the plasma AA profile and AA loss into the dialysate and having distinct compositions of administered AAs between the intervention and control groups [9–12]. In addition, nutritional status assessment by the malnutrition inflammation score (MIS) as a subjective global assessment (SGA)-based tool specifically for dialysis patients [18] after IDAA replacement has been barely explored. Herein, we conducted a randomized controlled study to compare the different techniques between the continuous infusion and acute load of similar intradialytic parenteral nutrition consisting of glucose and AA solution enriched with high branched-chain content to facilitate muscle metabolism on comprehensive nutritional outcomes, including serum albumin level, muscle quantity and muscle quality. We also investigated the changes of plasma and dialysate AA levels after receiving each technique of IDAA replacement.

## MATERIALS AND METHODS

### Study design and participants

This was a prospective, open-label, randomized controlled study registered in the Thai Clinical Trial Registry (TCTR20230401003) [19]. This study was approved by the Institutional Review Board of the Faculty of Medicine, Chulalongkorn University (IRB no. 0208/66). Written informed consent was obtained from each participant before enrolment. The eligible participants were >18 years of age, receiving chronic HD for at least 3 months, having mild PEW determined by a serum albumin level >3.5 g/dl [20] but <4.0 g/dl [21] despite 7-point SGA in category A or MIS ≤5 [1] and able to provide informed consent. The exclusion criteria included participants with a known history of abnormal AA metabolism, allergy to any component of parenteral nutrition, persistent plasma glucose >300 mg/dl, concurrently on any kind of dietary supplement, receiving immunosuppressive agents, uncontrolled infection, metastatic cancer, advanced cirrhosis, active heart failure, pregnancy or lactation, using metallic devices or undergoing limb amputations. The total study period was 3 months. The primary objective of this study was to compare serum albumin levels between the continuous infusion and acute load of IDAA replacement at the study completion. The secondary outcomes were the changes in muscle mass, strength, physical performance, plasma AA levels and AA loss into spent dialysate. From the studies of Smolle *et al*. [22] and Oguz *et al*. [10], it was found that IDAA replacement was associated with a significant increase in serum albumin levels in HD patients of 0.31 g/dl and 0.18 g/dl, respectively. To estimate the mean difference of the change in serum albumin level of >0.13 g/dl between two independent samples with a study power of 80%, α error of 0.05 and assumed dropout rate of 20%, the estimated sample size was 24 patients per group (total *n* = 48). A computer-generated block of four randomizations with equal allocation was used. The allocation was concealed using sequentially numbered, opaque and sealed envelopes.

### Data collection and AA replacement protocols

All laboratory tests were analysed before the dialysis session and standardized in our hospital central laboratory. All patients underwent standard HD for 4 hours using biocompatible high-flux dialyser at a blood flow rate of 300–350 ml/min and a dialysate flow rate of 500 ml/min with a maximal ultrafiltration rate of 13–15 ml/kg/session. Enrolled participants were randomized 1:1 to receive the continuous infusion or acute load of IDAA (control). All patients were replaced with the same parenteral nutrition of 50% glucose 100 ml (200 kcal; Able Medical, Bangkok, Thailand) followed by 7.2% AA solution 200 ml (57.6 kcal; Kidmin, Thai Otsuka Pharmaceutical, Bangkok, Thailand). The composition of the AA solution was enriched with essential, non-essential and conditionally essential AAs of 14.4 g with a high branched-chain AA content of 6.6 g (Supplementary Table S1). IDAA was delivered during dialysis by a disposable intravenous setup with infusion pump (M. Care Engineering, Nanthaburi, Thailand) that directly connected to a venous drip chamber. Infusion of each solution was begun after 15 minutes from the start of dialysis and steadily instilled at a constant rate (85 ml/h) until the end of session in the continuous infusion group. In the acute load group, IDAA was administered in the last hour of dialysis with an approximate infusion rate of 800 ml/h (Supplementary Table S2). Intradialytic hypotension was defined as a decrease in systolic blood pressure ≥20 mmHg or a reduction of mean arterial pressure ≥10 mmHg associated with symptoms [23]. Both groups received similar counselling by renal dietitians, such as adequate calorie and protein intake, as well as other standard care for HD patients during the entire study period. A 3-day diet record or recall was analysed using a national database program (INMUCAL) based on the Thai food composition table for nutritional calculation. Also, dietary protein intake was estimated by calculation of the normalized protein equivalent of total nitrogen appearance (nPNA). The presence of residual renal function was defined as urine volume >200 ml from 24-hour urine collection [24].

### Measurement of outcomes

Serum albumin level measurement, biochemical and body composition analysis were collected at baseline and the end of study (3 months). Serum albumin level measurement was performed from mid-week pre-dialysis venous blood using the Bromcresol Green method (Alinity C). The three compartmental whole-body bioelectrical impedance spectroscopy (BIS) model containing 50 frequencies, ranging from 5 to 1000 kHz, was employed for body composition measurement (Body Composition Monitor). Total-body muscle mass together with overhydration status were derived from the impedance data. Measurements were taken 15 minutes before mid-week dialysis in the supine position for practical reasons. Handgrip strength was assessed using a hydraulic hand dynamometer ***(***Jamar***).*** We defined low muscle strength or weakness as handgrip strength of <27 kg in men and 16 kg in women [25]. A timed usual pace of a marked 4-m course before dialysis was used for gait speed and the faster of two walks was used for analysis. We defined low physical performance or slowness as gait speed ≤0.8 m/s [25]. Fasting blood was drawn from both before and after IDAA replacement to measure plasma free AA concentrations at the time of the first use of dialyser. The dialysate outflow was also obtained using hourly continuous sampling with a designated port within the same session as the collection of the plasma AA profile [26]. All dialysate effluent was mixed together in a small container and 3 ml of fluid volume was collected and stored at 4–8°C for final analysis within the same date. Heparinized plasma and dialysate specimens were analysed for free AA levels using liquid chromatography tandem mass spectrometry (MassChrom, ChromSystems, Munich, Germany).

### Statistical analysis

We described patient characteristics using mean ± standard deviation (SD) or 95***%*** confidence interval ***(***CI***)*** for normally distributed variables or median ***(***IQR***)*** for non-normally distributed variables and proportions for categorical variables. We compared patients’ characteristics using chi-squared, unpaired *t*-test and Mann-Whitney U test as appropriate. Serum albumin levels and other parameters on study completion were compared between groups using unpaired *t*-test or Wilcoxon rank-sum test. The changes in outcomes from baseline in each group were compared using a paired *t*-test and signed-rank test as appropriate. The data were analysed by a blind investigator in intention-to-treat analysis. We conducted all analyses in Stata 15 ***(***StataCorp, College Station, TX, USA***)*** and *P* values <.05 were considered statistically significant.

## RESULTS

### Baseline demographic data of participants

**Figure 1:**
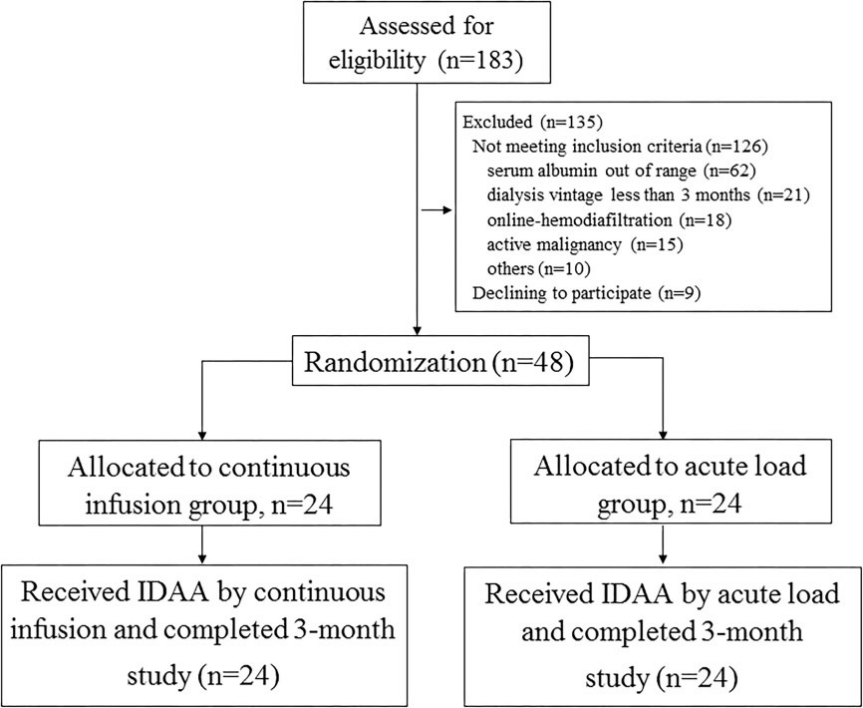
The Consolidated Standards of Reporting Trials diagram of the study.

A total of 183 dialysis patients were enrolled and 48 patients were finally randomized in this study (*n* = 24 per group) (Fig. 1). All of the participants completed the 3-month intervention period. The mean age of patients was 68.9 ± 12.7 years and 45.8% were men. The average serum albumin level at baseline was 3.75 ± 0.1 g/dl and did not differ between groups (*P* = .67). Demographic characteristics at baseline, including age, sex and insulin-treated diabetes patients, were not statistically different between the two groups (Table 1). All patients met the minimally required target of dialysis adequacy for small solute clearance and were classified as normal appetite determined by the 7-point SGA in category A. Overall, the mean MIS among participants was 6.4 ± 2.8 points, indicating a mild degree of PEW.

**Table 1:** Patient characteristics at baseline.

Parameters	Total (*N* = 48**)**	Continuous infusion group **(***n* = 24**)**	Acute load group **(***n* = 24**)**	*P*-value
Age (years)	68.9 ± 12.7	67.8 ± 12.5	69.9 ± 13.1	.56
Men, %	45.8	37.5	54.2	.25
Ischaemic heart disease, %	22.9	16.6	29.1	.30
Cerebrovascular disease, %	10.4	8.3	12.5	.64
Dialysis vintage (months), median (IQR)	36.0 (18.0–48.0)	36.0 (24.0–72.0)	24.0 (12.0–48.0)	.07
Tunnelled catheter, %	33.3	29.1	37.5	.54
Aetiology of ESKD, %				
Diabetic nephropathy	58.3	54.1	62.5	.55
Unknown	22.9	25.0	20.8	.73
Medication, %				
Insulin	18.8	8.3	29.1	.06
ACEI/ARB	14.6	12.5	16.6	.68
Body weight (kg)	58.1 ± 13.9	56.6 ± 12.8	59.6 ± 14.9	.46
SBP (mmHg)	145.1 ± 23.4	143.5 ± 26.4	146.7 ± 20.3	.64
DBP (mmHg)	74.8 ± 14.2	75.7 ± 14.9	73.9 ± 13.7	.66
Presence of RKF^a^, %	35.4	25.0	50.0	.06
Urine volume (ml), median (IQR)	500 (260–800)	800 (250–840)	450 (280–750)	.67
Urea reduction ratio (%)	79.8 ± 8.2	79.6 ± 7.8	80.6 ± 8.7	.90
Single pool Kt/V	2.0 ± 0.4	2.0 ± 0.4	2.1 ± 0.5	.74
Serum albumin (g/dl)	3.7 ± 0.1	3.7 ± 0.1	3.7 ± 0.1	.67
Blood urea nitrogen (mg/dl)	62.9 ± 16.0	61.5 ± 18.4	64.4 ± 13.4	.53
Serum creatinine (mg/dl)	9.3 ± 2.5	9.3 ± 2.4	9.2 ± 2.6	.90
Serum sodium (mg/dl)	136.7 ± 3.4	137.2 ± 3.0	136.2 ± 3.7	.26
Serum potassium (mg/dl), median (IQR)	4.3 (3.9–4.8)	4.3 (3.8–5.0)	4.4 (3.9–4.7)	.32
Serum bicarbonate (mg/dl)	23.3 ± 2.6	23.3 ± 3.1	23.3 ± 1.9	.99
Serum calcium (mg/dl)	8.9 ± 0.7	8.9 ± 0.6	8.8 ± 0.7	.60
Serum phosphate (mg/dl)	5.0 ± 1.8	5.3 ± 1.8	4.6 ± 1.7	.18
Haemoglobin (g/dl)	11.0 ± 1.2	11.1 ± 1.2	11.0 ± 1.1	.86
Haemoglobin A1C (%)	6.1 ± 1.0	5.9 ± 0.8	6.2 ± 1.2	.39
Fasting plasma glucose (mg/dl), median (IQR)	107.5 (85.0–149.0)	103.0 (84.0–149.0)	112.0 (87.0–149.0)	.77
LDL-C (mg/dl), median (IQR)	77.0 (63.0–109.0)	81.0 (63.5–113.0)	67.0 (63.0–95.0)	.26
Serum 25-(OH)D (ng/ml)	39.3 ± 9.0	40.6 ± 9.3	37.3 ± 9.0	.51
MIS in category C^b^, %	58.7	69.5	47.8	.13
MIS scores	6.4 ± 2.8	7.0 ± 2.8	5.8 ± 2.7	.16
Normalized PCR (g/kg/day)	1.2 ± 0.3	1.1 ± 0.3	1.2 ± 0.2	.75

Data are presented as mean ± SD unless stated otherwise.

ACEI: angiotensin-converting enzyme inhibitor; ARB: angiotensin receptor blocker; DBP: diastolic blood pressure; LDL-C, low-density lipoprotein cholesterol; PCR: protein catabolic rate; RKF, residual kidney function; SBP: systolic blood pressure; 25(OH)D: 25-hydroxyvitamin D.

*P-*values indicate a comparison between two groups at baseline.

a RKF defined as urine volume >200 ml/day.

b MIS category C defined as MIS ≥6.

### Effects of different AA replacement protocols on nutritional endpoints

**Figure 2:**
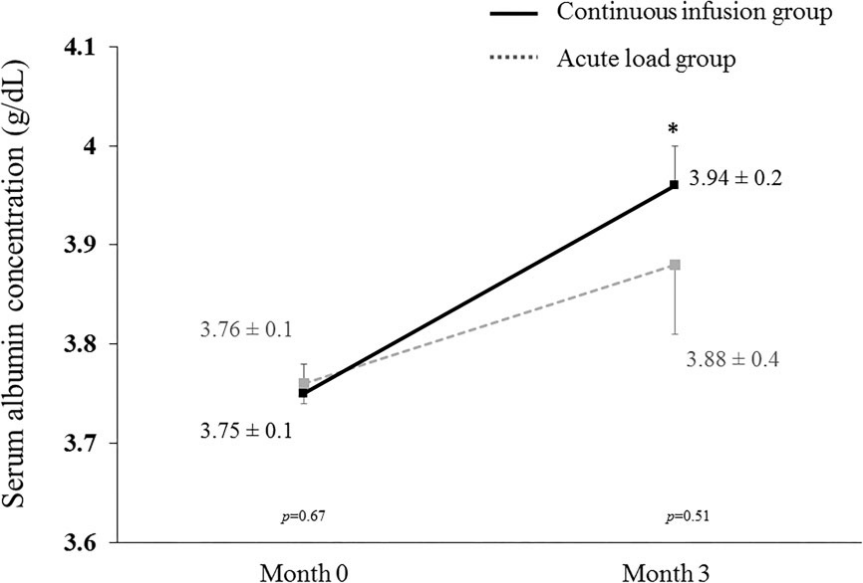
Comparison of serum albumin levels between continuous infusion and acute load techniques of IDAA replacement at baseline and 3 months. Data shown as mean ± SD. Error bars show standard error of the mean. *P*-values indicate the comparison between groups. *P < .05 after 3 months when compared with baseline values.

Nearly all patients (98.7%) were adherent to the scheduled administration of IDAA and 99.5% were in compliance with the prescribed parenteral nutrition volume for each HD session. Although the average serum albumin levels were not statistically different between the two groups at the end of 3 months (*P* = .51), the absolute serum albumin level was significantly elevated from baseline by 0.20 g/dl (95% CI 0.08–0.29) after 3 months of IDAA replacement in the continuous infusion group (3.75 ± 0.1–3.94 ± 0.2 g/dl; *P* = .001). In contrast, serum albumin levels were unaltered, with a mean difference of 0.11 g/dl (95% CI −0.04–0.27) in the acute load group at the end of 3 months (3.76 ± 0.1–3.88 ± 0.4 g/dl; *P* = .13) (Fig. 2). Despite comparable energy and protein intake, BIS-derived total body muscle mass indexed to height squared was also significantly increased in the continuous infusion group at 3 months (11.7 ± 2.9–12.8 ± 2.9 kg/m^2^; *P* = .03) while it remained unchanged in the acute load group (12.0 ± 2.8–12.3 ± 3.4 kg/m^2^; *P* = .45).

Although anthropometry including BMI and waist circumference was not increased from baseline and at the end of 3 months in both groups, MIS was significantly improved at the end of the study in the continuous infusion and acute load groups (Table 2). However, there were no significant changes in either muscle function measured by sex-specific handgrip strength or physical performance determined by gait speed after IDAA replacement from baseline and at the end of 3 months between the two groups (all *P* > .05). Those results were similar after stratification by categorical values into the presence of weakness and slowness in both the continuous infusion and loading protocol groups (Table 2).

**Table 2:** Comparison of changes of secondary endpoints and other nutrition-related parameters between groups.

	Continuous infusion group **(***n* = 24**)**			Acute load group **(***n* = 24**)**			
Parameters	Baseline	After 3 months	*P*-value^c^	Baseline	After 3 months	*P*-value^d^	*P*-value^b^
Anthropometry and body composition							
Body mass index (kg/m^2^)	22.3 ± 4.4	22.6 ± 4.5	.28	23.3 ± 4.3	23.3 ± 4.4	.93	.56
Waist circumference (cm)	87.4 ± 13.8	88.2 ± 13.9	.10	87.2 ± 13.2	88.0 ± 13.5	.11	.96
Total body lean tissue (kg)	29.4 ± 8.3	31.8 ± 8.8	.08	30.8 ± 8.6	31.6 ± 10.4	.43	.96
Total body lean tissue/height^2^ (kg/m^2^)	11.7 ± 2.9	12.8 ± 2.9^c^	.03	12.0 ± 2.8	12.3 ± 3.4	.45	.59
Excess extracellular fluid (L)	2.9 ± 1.9	2.6 ± 1.5	.32	2.7 ± 1.3	2.8 ± 1.4	.67	.67
3-day dietary record							
Energy intake (kcal/kg/day)	20.4 ± 5.3	19.4 ± 4.6	.89	18.1 ± 5.1	18.3 ± 4.8	.49	.40
Protein intake (g/kg/day)	0.9 ± 0.2	0.8 ± 0.2	.07	0.8 ± 0.3	0.7 ± 0.2	.28	.78
Muscle function and physical performance							
Handgrip strength (kg)	14.7 ± 6.9	13.7 ± 6.5	.12	15.4 ± 8.1	15.8 ± 7.5	.48	.31
Men	18.8 ± 6.6	17.4 ± 5.5	.32	21.7 ± 5.6	21.5 ± 5.6	.86	.11
Women	12.3 ± 6.2	11.4 ± 6.2	.27	8.5 ± 3.2	9.6 ± 3.3	.16	.38
Gait speed (m/s)	0.8 ± 0.3	0.9 ± 0.3	.12	0.8 ± 0.3	0.9 ± 0.3	.67	.68
Presence of weakness^a^, %	79.2	83.3	.07	87.5	91.3	.11	.41
Presence of slowness^a^, %	47.0	33.3	.10	55.6	47.0	.06	.43
Other laboratory data							
Fasting plasma glucose (mg/dl)	122.6 ± 42.6	140.0 ± 43.4	.08	121.7 ± 43.4	119.8 ± 36.2	.91	.84
Haemoglobin A1C (%)	6.0 ± 0.9	6.1 ± 0.8	.67	6.3 ± 1.2	5.9 ± 0.9	.05	.12
Serum calcium (mg/dl)	8.9 ± 0.7	8.6 ± 1.0	.23	8.8 ± 0.7	8.7 ± 0.8	.87	.69
Serum phosphate (mg/dl)	5.3 ± 1.8	4.6 ± 1.9	.06	4.6 ± 1.7	4.9 ± 2.3	.46	.54
Serum bicarbonate (mg/dl)	23.3 ± 3.1	23.7 ± 3.2	.53	23.4 ± 1.9	22.9 ± 2.7	.38	.36
Single-pool Kt/V	2.0 ± 0.4	1.9 ± 0.4	.17	2.1 ± 0.5	1.8 ± 0.5	.05	.86
Urea reduction ratio (%)	79.6 ± 7.8	78.3 ± 8.1	.29	80.6 ± 8.7	78.4 ± 9.2	.06	.97
MIS	7.0 ± 2.8	6.0 ± 2.6^c^	.004	5.8 ± 2.7	5.1 ± 2.8^d^	.02	.26
Normalized PCR (g/kg/day)	1.1 ± 0.3	1.1 ± 0.4	.35	1.2 ± 0.2	1.1 ± 0.3	.31	.85

Data are presented as mean ± SD.

PCR: protein catabolic rate.

a Weakness defined as handgrip strength <27 kg in men and <16 kg in women and slowness defined as gait speed ≤0.8 m/s.

b *P*-value between groups comparison at month 3.

c *P* < .01 after 3 months in the continuous infusion group compared with baseline.

d *P* < .05 after 3 months in the acute load group compared with baseline.

### Plasma AA concentrations and AA losses between two infusion techniques

All basal pre-dialysis plasma AA levels were not statistically different between the two groups (all *P* > .05). The sum of post-dialysis total plasma AA levels (*P* = .57) consisting of both essential (*P* = .59) and non-essential AAs (*P* = .66) did not significantly differ between both groups. For analysis of median individual AA concentrations after IDAA replacement at post-dialysis, plasma phenylalanine (*P* = .001), methionine (*P* = .01), tyrosine (*P* = .001) and arginine (*P* = .001) were significantly higher in patients receiving the acute load than continuous infusion technique. However, post-dialysis plasma branched-chain AA levels at the end of dialysis, including leucine and valine, were comparable between the continuous infusion and acute load groups (Table 3).

**Table 3:** Plasma amino acid levels between different infusion techniques.

	Continuous infusion group **(***n* = 24)			Acute load group **(***n* = 24)			
Plasma amino acid levels (µmol/l)	Pre-dialysis	Post-dialysis	*P-*value^d^	Pre-dialysis	Post-dialysis	*P-*value^d^	*P*-value^c^

Essential amino acids	1448.6 (1051.5–2179.1)	1342.3 (86.9–1804.8)^d^	<.001	1350.7 (980.6–1870.6)	1398.6 (1143.9–1969.8)	.53	.59
Leucine^b^	1138.0 (824.0–1998.0)	975.5 (602.0–1412.5)^d^	.002	990.5 (774.5–1599.5)	885.0 (635.0–1442.0)^d^	.03	.61
Valine^b^	199.0 (155.5–237.5)	204.0 (174.0–251.5)	.30	182.5 (162.5–234.5)	268.5 (236.5–379.0)	.06	.09
Tyrosine^a^	46.8 (39.9–53.4)	37.4 (28.5–42.3)^d^	.001	48.1 (39.8–60.4)	45.2 (40.1–58.9)	.27	.001
Phenylalanine	43.2 (35.0–47.9)	66.1 (50.6–84.3)^d^	<.001	46.7 (38.2–61.9)	101.5 (74.9–115.0)^d^	<.001	.001
Methionine	10.8 (9.5–13.4)	16.7 (10.1–20.2)^d^	.006	11.1 (9.0–15.4)	21.5 (14.2–29.4)^d^	<.001	.01
Non-essential amino acids	1760.3 (1432.2–2236.7)	1578.2 (1166.3–1736.1)^d^	<.001	1664.6 (1519.4–1719.9)	1856.3 (1394.9–1663.5)	.37	.66
Alanine	350.8 (273.5–670.0)	322.5 (233.3–564.0)^d^	.04	321.0 (263.5–398.0)	312.5 (258.0–411.5)	.58	.14
Arginine	25.4 (22.7–28.6)	21.8 (14.9–26.4)	.12	30.1 (23.3–43.4)	33.9 (27.0–51.1)	.09	.001
Aspartic acid	257.5 (227.0–292.0)	224.5 (182.0–272.5)^d^	<.001	243.5 (180.5–264.5)	240.5 (180.5–292.5)	.18	.12
Proline	208.5 (156.5–290.5)	155.5 (124.5–207.5)^d^	<.001	191.0 (158.5–232.5)	169.5 (136.5–204.5)^d^	.01	.61
Glutamic acid	345.5 (297.0–421.0)	300.0 (253.5–379.0)^d^	.008	326.0 (284.0–395.5)	313.5 (276.5–390.5)	.30	.73
Glycine	196.5 (149.5–234.5)	155.0 (121.0–229.5)	.25	198.0 (122.0–244.5)	172.0 (123.3–219.0)	.06	.66
Citrulline	93.1 (82.9–118.5)	65.3 (49.6–80.3)^d^	<.001	102.5 (88.0–124.0)	68.6 (62.2–78.4)^d^	<.001	.54
Ornithine	116.5 (108.0–105.0)	92.8 (74.7–116.0)^d^	<.001	112.5 (103.5–144.0)	104.5 (89.7–123.0)^d^	<.002	.44
Total amount of amino acids	3468.1 (2462.9–4261.8)	3039.4 (2028.0–3495.8)^d^	<.001	2999.8 (2582.6–3533.9)	3132.7 (2553.8–3653.1)	.92	.57

All basal pre-dialysis plasma AA levels were not statistically different between two groups (all *P* > .05).

a Conditionally essential amino acid in kidney disease patients.

b Branched-chain amino acids.

c *P*-values indicate a comparison of post-dialysis plasma amino acid levels between continuous infusion and acute load groups.

d *P* < .05 compared post-dialysis with pre-dialysis values within the same group.

When compared with pre-dialysis plasma concentrations, almost all individual essential and non-essential AAs decreased after IDAA administration by the continuous infusion technique (all *P* < .05). In contrast, the majority of each post-dialysis plasma AA concentration was sustained relative to the pre-infusion value before dialysis in the acute load group.

**Figure 3:**
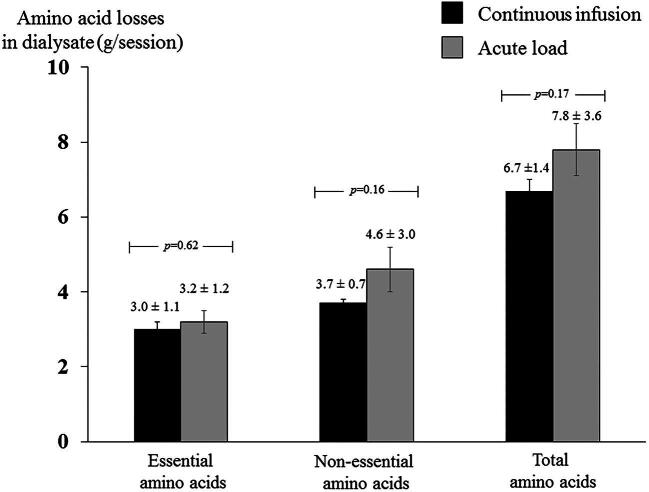
Comparison of essential, non-essential and total amount of AA loss into the dialysate between the continuous infusion and acute load groups of IDAA replacement. Data shown as mean ± SD. *P*-values indicate the comparison between groups.

Although there was a statistically significant loss of glycine in the acute load compared with continuous infusion group (0.38 ± 0.1 versus 0.31 ± 0.1 g/session; *P* = .02), the average total amount of AA losses into the dialysate were not different between the two groups (*P* = .17). These comparable results regarding AA losses in both the continuous infusion and acute load groups persisted when analysis was stratified by essential and non-essential components of AA (Fig. 3).

### Reported adverse events

None of the patients receiving IDAA replacement had worsening metabolic acidosis, decreased urea reduction ratio and dialysis inadequacy (Table 2). Neither volume overload nor uncontrollable dysglycaemia was found throughout the entire study period. There were no significant differences in intradialytic hypotension episodes between the continuous infusion and acute load groups (4.0 versus 5.4 episodes per 100 HD sessions; *P* = .29). None of the patients receiving IDAA by continuous infusion was reported for any adverse events, while two patients (8.3%) in the loading protocol group experienced headache and abdominal discomfort (Table 4).

**Table 4:** Reported adverse effects of different IDAA infusion techniques during the study period.

Characteristics	Continuous infusion group (*n* = 24)	Acute load group (*n* = 24)	*P*-value
Intradialytic hypotension^a^, *n*	4.0	5.4	0.29
Volume overload, *n*	0	0	NA
Hypoglycaemic events, *n*	0	0	NA
Manageable hyperglycaemia, *n*	0	1	0.99
Headache, *n*	0	2	0.48
Abdominal discomfort, *n*	0	2	0.48
Nausea and/or vomiting, *n*	0	1	0.99
Skin rash, *n*	0	0	NA
Death, *n*	0	0	NA

NA: not applicable.

*P*-values >.05 indicate no statistical significant difference between groups.

a Defined as a decrease in systolic blood pressure ≥20 mmHg or a reduction of mean arterial pressure ≥10 mmHg associated with symptoms and calculated per 100 haemodialysis sessions.

## DISCUSSION

The study presented here demonstrates that serum albumin level was significantly increased after a 3-month IDAA replacement with the continuous infusion technique. A significant improvement of BIS-derived total body muscle mass indexed to height squared was also observed after continuous infusion of IDAA for 3 months. Both total post-dialysis plasma AA concentrations and the amount of AA losses into dialysate were comparable between the continuous infusion and acute load groups. The measurements of muscle strength and gait speed were not significantly different between the two techniques of IDAA replacement.

Inadequacy of oral dietary protein intake among HD patients with PEW predominantly causes a decrease in the rate of albumin synthesis, through a reduction in the availability of AAs, while fractional catabolism of albumin continues at a normal rate. As a result, a gradual decrease in the concentration of plasma albumin level occurs without exogenous AA replacement [27]. Given that the average baseline BMI of participants in the present study was approximate to overweight criteria among the Asian population [28], nutritional support by oral nutritional supplement (ONS), which generally contains an energy-dense high protein content to help enhance albumin synthesis, might lead to an increase in the prevalence of obesity. To mitigate the lack of adherence and low compliance associated with ONS use over time, IDAA may be considered as an alternative with reasonable costs and benefits among HD patients [8]. However, there is no consensus on how best to deliver parenteral nutrition containing AAs during dialysis. A previous study by Heidland *et al*. [9] observed that AAs administration by intravenous loading near the end of each HD session was significantly associated with an increase in serum albumin level after 16 weeks. However, no control group was available for comparison and the recruited participants were inhomogeneous in terms of protein-energy status in their study [9]. Although IDAA by the loading technique also revealed a significant increase in serum albumin concentration in a comparative study conducted by Oguz *et al*. [10], the control and intervention groups received different routes and dissimilar doses and compositions of infused AAs. In agreement with our findings and using an AA dose calculation based on the patient's body weight and no provision of glucose before each AA infusion, a non-randomized study by Smolle *et al.* [22] showed that IDAA replacement using continuous infusion over the entire session of dialysis for 16 weeks resulted in a significant improvement in serum albumin concentration among maintenance HD patients. Additionally, our present findings were supported by a previous experimental study of Navarro *et al*. [11] among non-diabetic HD patients showing that the average serum albumin level was significantly increased from baseline after IDAA replacement by the continuous infusion technique compared with the control subjects without AA supplementation.

We found that the average increment of serum albumin level, as one of the best predictors of mortality among ESKD patients [29], with IDAA using continuous infusion in this study was 0.2 g/dl. Such impact may lead to considerable improvements in mortality and hospitalization among the HD population [30]. Furthermore, BIS-derived total body muscle mass indexed to height squared was also significantly increased from baseline in the continuous infusion group, while BMI, energy and protein intake were unchanged. In parallel with our study, a relatively large, multicentred study by Czekalski *et al*. [31] also indicated a small yet significant increase of mid-arm circumference, as a muscle mass surrogate, from baseline after continuous IDAA replacement since the start of dialysis. Although there was an increasing trend in serum albumin level and muscle mass in the acute load group, the findings did not reach statistical significance. We hypothesized that these observations might be partly explained by branched-chain AAs related to muscle formation, decreased intramuscular AAs release and increased utilization of plasma free AAs as substrate for visceral protein synthesis. Apart from plasma leucine and valine levels, other individual essential AA concentrations at post-dialysis (phenylalanine, methionine and tyrosine) were significantly lower among patients receiving IDAA by continuous infusion than the acute load technique (Table 3). Several previous studies [32–35] consistently revealed that plasma branched-chain AAs, particularly leucine, which has been shown as a key metabolite in skeletal muscle protein synthesis [36], were deficient among malnourished HD patients when compared with healthy control subjects. Therefore, the restoration of plasma branched-chain AAs in relation to other essential AA levels in patients receiving continuous IDAA infusion is likely to be associated with the attenuation of muscle catabolism observed during HD treatment. When compared with pre-dialysis plasma values, patients receiving IDAA by continuous infusion had significantly lower levels for nearly all post-dialysis plasma total and individual AA concentrations. A study by Fujiwara *et al*. [37] showed that HD-related cell starvation, which occurs to a lesser extent among the non-diabetic HD population compared with their diabetic counterparts, typically begins at the first hour of HD and reaches the plateau phase during the third hour of the session. This particular occurrence is accompanied by cellular energy depletion due to gluconeogenesis suppression followed by the release of free AAs from muscle protein breakdown into the blood circulation for compensatory energy production. Accordingly, we consider that the co-administration of IDAA along with glucose since the start of the HD session may alleviate this process, as indicated by the decrease in the individual plasma AA level at post-dialysis compared with pre-dialysis values, and finally leads to not only the acceleration of albumin synthesis, but also muscle mass improvement.

A former objection against IDAA by continuous infusion was speculation that the infused AA may be lost during the dialysis process. However, our results revealed that there was no significant difference in the average essential, non-essential and total amount of AA losses into the dialysate between the two groups. A previous study by Wolfson *et al*. [12] revealed only a slight increase in the loss of free AAs into the dialysate (2-3 g per session) when compared with normal saline as a control and the large preponderance (≈80%) of infused AAs were retained despite using continuous infusion since the start of HD. Although using on-line haemodiafiltration instead of conventional HD and having much smaller number of participants, findings from a recent observational study by Kato *et al*. [13] among HD patients were in agreement with ours, that the amount of both essential and non-essential AA leakage into the spent dialysate were not significantly different between continuous infusion of IDAA and the loading technique at the last hour of the HD session. Given that the rapid transfusion of IDAA by the acute load technique near the end of dialysis might be associated with an abrupt increase in plasma AA levels over a short period of time, these undesirable out-of-value AAs in the blood circulation might result in a blunt and inefficient metabolic utilization of nutrients [38]. Due to the fact that the infused AAs are dialysable, these sustained non-physiologically high plasma AA levels by acute load might also pass through the high-flux membrane and possibly yield a greater loss of free AAs into the dialysate during the last hour of HD relative to continuous infusion, resulting in the comparable dialysate loss of AAs between the two IDAA infusion techniques in our study.

Certain strengths of this study should be mentioned. The present randomized controlled study is the first to compare the performance of IDAA replacement by using different infusion techniques on comprehensive nutritional outcomes together with the dynamics of plasma AA concentrations and dialysate loss of free AAs. We intended to utilize MIS to assess the nutritional status among HD patients. Moreover, we used BIS instead of anthropometry to minimize the influence of fluid overload for estimation of muscle mass among the HD population [29]. Despite appropriate statistical calculations, the number of patients in our study is still relatively low. We did not collect mid-session AA levels to detect plasma AA kinetics throughout the whole process of the dialysis session. We did not measure AA levels in other compartments such as red blood cells and muscle, which have also been disturbed in normal metabolism among HD patients with PEW [39]. Lastly, the effect of serum albumin improvement utilizing continuous infusion of IDAA in our study was observed in the conventional HD modality with no concurrent loss of albumin during dialysis treatment [40]. Additional research with a greater number of participants is needed to determine whether IDAA replacement using continuous infusion in other extracorporeal blood purification techniques, such as convective therapy with highly permeable membranes, leads to sustained improvement in the serum albumin level as well as muscle health among the HD population.

In conclusion, IDAA replacement using the continuous infusion technique appears to be superior to the acute load in terms of an improvement in serum albumin levels, a survival surrogate among HD patients. A significant improvement in muscle quantity, but not muscle quality, was also observed after continuous infusion of IDAA. Both total post-dialysis plasma AA concentrations as well as dialysate AA loss were similar between the two techniques of IDAA replacement.

## Supplementary Material

sfae361_Supplemental_File

## Data Availability

The datasets generated and/or analysed during the current study are not publicly available due to the Personal Data Protection Act, B.E.2562 (2019), but are available from the corresponding author upon reasonable request.
