# Supplementary material for: Nutritional outcomes between different techniques of intradialytic amino acid replacement: a randomized controlled trial
Source: Clin Kidney J. 2024 Nov 18;18(1):sfae361. doi: 10.1093/ckj/sfae361 (PMC11852333; doi:10.1093/ckj/sfae361)
Supplement: sfae361_Supplemental_File [file sfae361_Supplemental_File.docx]

**Supplemental Table S1** Composition of parenteral amino acid solution (Kidmin^®^)

| **Amino acid** | **Each 200 mL** |
| --- | --- |
| L-Leucine  L-Isoleucine  L-Valine  Lysine acetate (L-Lysine equivalent)  L-Threonine  L-Tryptophan  L-Methionine  L-Phenylalanine  L-Cysteine  L-Tyrosine  L-Arginine  L-Histidine  L-Alanine  L-Proline  L-Serine  L-Aspartic Acid  L-Glutamic Acid  Water for Injection  Total free amino acids  Essential amino acids (E)  Non-essential amino acids (N)  E/N ratio  Branched-chain amino acids  Total Nitrogen | 2.80 g  1.80 g  2.00 g  1.42 g (1.01 g)  0.70 g  0.50 g  0.60 g  1.00 g  0.20 g  0.10 g  0.90 g  0.70 g  0.50 g  0.60 g  0.60 g  0.20 g  0.20 g  ad 200 mL  14.41 g  10.41 g  4.00 g  2.6  45.8 % (w/w)  2.00 g |

**Supplemental Table S2** Comparison of the total amount of intradialytic glucose and amino acids replacement per each hemodialysis session between continuous infusion and acute load groups

| **Composition** | **Continuous infusion group (n=24)** | **Acute load group (n=24)** |
| --- | --- | --- |
| Total amino acids, g  Total amino acids, kcal  Total glucose, g  Total glucose, kcal  Total energy, kcal  Total volume of glucose and amino acids, ml | 14.4  57.6  50  200  257.6  300 | 14.4  57.6  50  200  257.6  300 |
